# Ring-opening copolymerization thermodynamics and kinetics of γ-valerolactone/ϵ-caprolactone

**DOI:** 10.1371/journal.pone.0199231

**Published:** 2018-06-21

**Authors:** Mariacristina Gagliardi, Angelo Bifone

**Affiliations:** 1 Center for Micro Bio-Robotics @SSSA, Istituto Italiano di Tecnologia, viale Rinaldo Piaggio, 34, 56025, Pontedera, Italy; 2 Center for Neuroscience and Cognitive Systems @UNITN, Istituto Italiano di Tecnologia, Corso Bettini 31, 38068 Rovereto, Italy; Queen’s University Belfast, UNITED KINGDOM

## Abstract

The general misconception that *γ*-lactones are not thermodynamically polymerizable has limited the development of all *γ*-lactone-based copolymers. A few studies have reported copolymerization of these five-membered cyclic esters with more reactive monomers, yet a systematic investigation of kinetics and thermodynamics is still lacking. To explore the feasibility of the reaction, we combined equilibrium and non-isothermal syntheses for the copolymerization of *γ*-valerolactone with *ϵ*-caprolactone, initiated with methoxy polyethyleneglycol and catalyzed by Tin(II) 2-ethylhexanoate. Here, we present the polymerization kinetic and thermodynamic parameters for different monomer ratios in the reaction feed. We observed the dependency of enthalpy and entropy of polymerization upon monomer ratio changes, and estimated a linear increase in the activation energy by increasing the *γ*-valerolactone fraction in the starting monomer mixture. Our data demonstrate that *γ*-valerolactone can copolymerize with *ϵ*-caprolactone, but only under specific conditions. The reaction parameters determined in this study will enable preparation of additional *γ*-valerolactone-based copolymers and development of a family of degradable materials with improved properties in respect to commonly used polyesters.

## Introduction

Degradable polymers are attracting increasing attention for their application in the fields of green chemistry, food packaging, agriculture and nanomedicine [1]. Hydrolyzable materials are particularly interesting for their short half-life, complete degradation and favorable clearance of degradation products. Aliphatic polyesters are the most widespread hydrolizable polymers due to their favorable characteristics [2], and cyclic esters lactones are starting reactants for their synthesis.

The majority of short-chain (four-, six- and seven-membered) cyclic esters are useful monomers that easily polymerize via Ring-Opening Polymerization (ROP). The ability of lactones to react via ROP is determined by the ring stability and their aptitude to shift the monomer/polymer equilibrium to the product side.

Ring strain energy is directly related to the thermodynamic stability of cyclic monomers. Ring strain energy derives from several contributions, including angular strain, bond stretching, compression or torsion, conformational strain due to repulsion between eclipsed hydrogen atoms, and transannular strain generated by interactions between side substituents. Previous studies evaluated the ring strain energy by varying the number of ring members [3], and associated lower energy values to five-membered lactones, with small differences for substituted (e.g. *γ*-valerolactone (GVL)) or unsaturated (e.g. *α*-angelica lactone) rings. Some rings show similar strain energy values, like *γ*-valerolactone and glycolide [4], but their reactivity in ROPs is completely different. Thus, in the analysis of lactone ROP also the thermodynamic aspect has to be considered.

Similarly to all chemical reactions, lactone polymerization proceeds until the equilibrium concentration of unreacted monomers is reached. According to thermodynamics, monomer polymerizability is related to the free Gibbs energy of polymerization (Δ*G*_*p*_) that depends on the polymerization enthalpy (Δ*H*_*p*_) and entropy (Δ*S*_*p*_). Lactone thermodynamic parameters highlight a dependency on member number, in particular for five-membered *γ*-lactones [5]. In lactones, Δ*H*_*p*_ is the main contribution to Δ*G*_*p*_ in small rings (3- or 4-membered) while Δ*S*_*p*_ does not significantly vary up to 6-membered molecules. In larger rings, enthalpy and entropy contribute equally to the free Gibbs energy of polymerization. Values of Δ*H*_*p*_ and Δ*S*_*p*_ lead to negative Δ*G*_*p*_ for all lactones in except of 5-membered rings [4].

**Fig 1:**
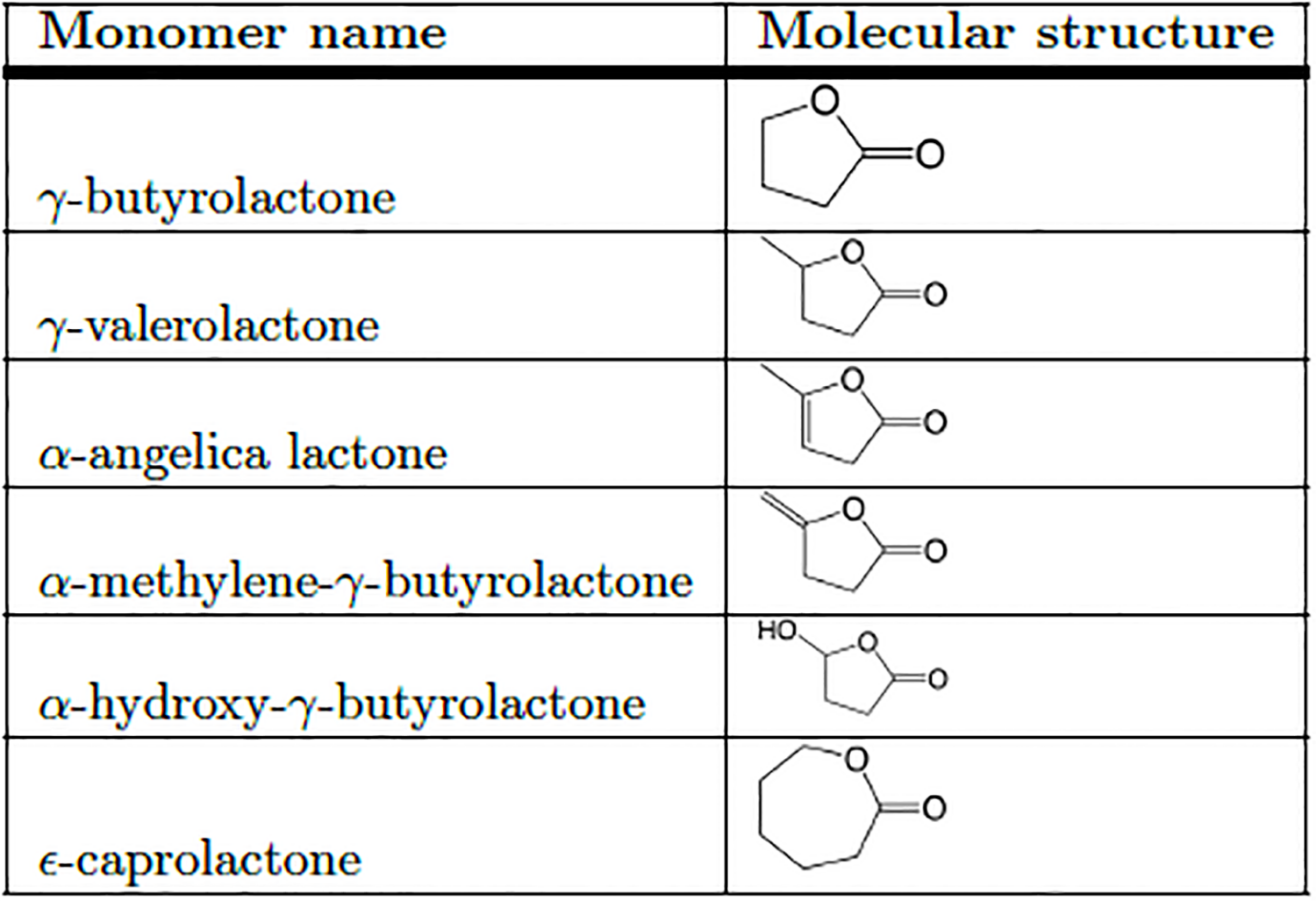
Molecular structures of cited lactones.

*γ*-butyrolactone (GBL), the simplest five-membered cycle, presents three methylenes between O and C = O. GBL has been long thought to be non-polymerizable [6], if not under drastic conditions [7], with dangerous catalysts [8], or after molecular modifications [9]. Although a certain number of homo- or copolymers of the GBL were reported in the literature [6, 10–13], this misconception has probably delayed the polymerization of *γ*-lactone family (Fig 1). Only recent studies reported the use of substituted *γ*-lactones, like *α*-methylene-*γ*-butyrolactone and its derivatives, in radical copolymerization with methyl methacrylate and styrene [14], for which reaction rates and yields are comparable to those of acrylic monomers.

*γ*-lactones were already used as co-monomers in the synthesis of copolymers containing *ϵ*-caprolactone [8, 15]. The *α*-hydroxy-*γ*-butyrolactone monomer was recently used as co-monomer in polyester synthesis, to provide additional -OH substituents for branching chain growth. Another interesting GBL derivative is *γ*-valerolactone, which brings an exocyclic methyl group. Recently, the authors reported the GVL copolymerization with *ϵ*-caprolactone (ECL, Fig 1) to give polyesters with improved hydrophilicity and hydrolytic degradation kinetics [16]. Improved functional properties derived from the presence of the substituent methyl group that significantly modified molecule arrangement. Major effects of the 4-hydroxyvalerate (4HV) repeating unit are: regulation of cristallinity [17], change of mechanical properties, inducing a decrease of material stiffness [18], and reduction of enzymatic degradation [19]. GVL units also promotes a safe degradation, enabling the preparation on highly cytocompatible nanoparticles for intracellular drug delivery [20].

Thermodynamics and kinetics for lactone ROP are only limited to the most common monomers, like lactide [21, 22]. Available thermodynamic and kinetic data on lactone ROPs were thoroughly reviewed by Duda and co-workers [5, 23]. They collected values of polymerization entropy and enthalpy for different-membered lactones, and indicated values of equilibrium monomer concentration. Quantum mechanics calculations showed that the thermodynamic polymerizability of six- and seven-membered lactones is similar, and confirmed unfavorable endergonic polymerizability of the GBL monomer [24], which is attributed to the low ring strain [25]. Additional thermodynamic data, provided by Schneiderman and Hillmyer, demonstrated the effect of side-groups on six-membered lactones [26].

To contribute in this field, we evaluated thermodynamics and kinetics for the copolymerization of GVL with ECL, initiated with methoxy polyethyleneglycol (mPEG) and catalyzed by Tin(II) 2-ethylhexanoate (Sn(Oct)_2_). To estimate thermodynamic parameters, we performed the equilibrium synthesis of three different copolymer compositions at six different temperatures. Experimental data provided values of Δ*H*_*p*_ and Δ*S*_*p*_. Kinetic parameters were computed by experimental data from non-isothermal polymerization at five different heating rates, while major details on copolymerization kinetics were obtained following ROP in a low-monomer-conversion range. Results demonstrated that the presence of GVL influenced the polymerization, and copolymerization can occur only within a specific range of feed compositions and working temperature.

## Materials and methods

### Materials

*ϵ*-caprolactone (ECL), *γ*-valerolactone (GVL), methoxy polyethyleneglycol (mPEG, Mn: 550 Da, initiator) and Tin(II) 2-ethylhexanoate (Sn(Oct)_2_, catalyst) were purchased from Sigma Aldrich. ECL and mPEG were dried at 60°C for 6 h and stored over molecular sieves before use, GVL and Sn(Oct)_2_ were used as received. The molar ratio between mPEG and monomer units was adjusted to obtain a theoretical molecular weight of 10 kDa. The amount of catalyst was in ratio 1/1 with mPEG. Molar monomer ratio ECL/GVL of the starting monomer mixture was varied (90/10, 80/20 and 70/30) to obtain three copolymer compositions. Homopolymer 100/0 was synthesized as reference. Monomer mixtures with lower ECL/GVL ratios and the reference 0/100 gave after ROP only oligomers (*M*_*n*_ lower than 1000 Da), and were not considered for this study.

### Analytic methods

The GPC apparatus was composed of a Shimadzu Prominence UFLC chromatograph, two ResiPore/PLGel GPC column (Agilent Technology, Italy), a tetra-detector equipped by UV diode array, refractive index, dual-angle light scattering (15° and 90°) and viscometer. Columns and detectors were thermostatted at 40°C. THF (Sigma Aldrich, Chromasolv^®^, 1 mL min^-1^) was used as eluent. Samples for GPC analysis were dissolved in THF (concentration: 0.1–0.2 mg mL^-1^, injection volume: 50 μL). Data analysis was based on a calibration curve obtained with narrow polystyrene standards.

NMR spectra were acquired on polymer solutions in CDCl_3_ (Aldrich) with a Bruker 400 MHz spectrometer (Bruker, Italy) at fixed temperature (300 K). Polymers for ^1^H-NMR analysis were dried under vacuum, for at least 24 h, before their solubilization in CDCl_3_ (0.2 mg mL^-1^).

FTIR analysis was conducted on polymer powders with a Shimadzu IRAffinity-1 spectrometer.

Non-isothermal polymerization was performed in a DSC1 apparatus (Mettler Toledo, Italy). The instrument was calibrated before each analysis session [27]. Polymerization was obtained under dry N_2_ flow (80 mL min^-1^) at different heating rates (5, 10, 15, 20 and 30 K min^-1^) in the temperature range from 300 to 525 K.

### Ring-opening polymerization procedures

Equilibrium and low-conversion polymerizations were performed in 10 mL one-neck round-bottom glass reactors. mPEG (1.5⋅10^−4^ mol) and liquid monomers (Table 1) were transferred in the reactor, flushed with dry N_2_ for 15 min, then stirred and heated at fixed temperature. The catalyst Sn(Oct)_2_ (1.5⋅10^−4^ mol, dissolved in 50 μL of toluene) was added upon heating. The reactor was connected to a CaCl_2_ column and maintained under N_2_ flux. Small amounts of crude products were withdrawn and immediately dissolved in cold acetone to stop the reaction. Crude samples were maintained at −20°C before the GPC analysis, then evaporated, weighted and dissolved in THF. Eventually, the reactor was quenched in cold water, products were dissolved in acetone, precipitated in methanol three times and finally dried under vented oven overnight at room temperature.

**Table 1:** Amounts of monomer used for reactions and molar composition of final products.

Monomer mixture (ECL/GVL)	ECL (mol)	GVL (mol)	Final products^a^ (ECL/GVL) (mol mol^−1^)
100/0	1.24 ⋅10^−2^	0.00	100/0
90/10	1.12 ⋅10^−2^	1.29 ⋅10^−3^	90/10
80/20	9.95 ⋅10^−3^	2.58 ⋅10^−3^	82/18
70/30	8.70 ⋅10^−3^	3.87 ⋅10^−3^	73/27

^a^ From ^1^H-NMR analysis; ratios between the linear counterparts of ECL and GVL in the final copolymers were determined on peak integrals of signals at 4.06 ppm and 2.37 ppm.

For equilibrium polymerizations, we performed six experiments, at 80, 90, 100, 110, 120 and 130°C. Reaction times were 36, 30, 24, 18, 12 and 6 h respectively [28]. For low-conversion reactions, ROPs were obtained at a fixed temperature (120°C). Samples were withdrawn after 15, 30, 45, 75, 120, 180, 240 and 300 min. For non-isothermal DSC polymerization, liquid monomer blends were freshly prepared before the analysis; 10 ± 2 mg of blend were weighted and added to sealed Al capsules.

Molecular weights for equilibrium, low-conversion and non-isothermal ROPs are summarized in Tables A, B and C in S1 File, respectively.

^1^H-NMR (Fig A in S1 File, 400 MHz, CDCl_3_): *δ* / ppm: 4.06 (-C**H**_2_COO-, dd, J = 8.6, 4.8 Hz, 2H), 3.64 (-OC**H**_2_CH_2_O-, s, 2H), 3.38 (C**H**_3_O- s, 3H), 2.30 (-OCOC**H**_2_CH_2_-, t, J = 7.5 Hz, 2H), copolymers showed an additional signal due to GVL at 2.37 ppm (-CH_2_CH(C**H**_3_)O-, t, J = 6.1 Hz, 3H). ^13^C-NMR (Fig B in S1 File, 400 MHz, CDCl_3_): *δ* / ppm: 173.60 (-O-**C**O-CH_2_-), 64.24 (-(CH_2_)_2_-O-, -CH_2_-(CH_2_)_3_-**C**H_2_-O-), 34.32 (-**C**H_2_-(CH_2_)_3_-CH_2_-O-), 28.56 (-CH_2_-CH_2_-**C**H_2_-CH_2_-CH_2_-), 25.74 (-CH_2_-CH_2_-CH_2_-**C**H_2_-CH_2_-), 24.77 (-CH_2_-**C**H_2_-CH_2_-CH_2_-CH_2_-), 21.40 (-CH_2_-CH_2_-C(**C**H_3_)-)).

FTIR / cm^-1^: 3600-3400 (O-H stretching), 3040-2730 (-CH- stretching), 1820-1600 (C = O ester bond stretching), 1420 (-CH_3_ bending, due to GVL), 1392 (C-O-H bending), 1210-1118 (CO-O ester bond stretching), 1070-1015 (C-O-C due to mPEG stretching), 720-675 (aliphatic -CH_2_- bending).

### ROP thermodynamics

Values of [*M*]_*eq*_ were quantified from GPC chromatograms of crude samples from equilibrium polymerization. Due to their similar molecular weight, ECL and GVL monomers were eluted together, allowing only the quantification of the overall concentration. The quantification was based on the coefficient of RI response *β*_*i*,*j*_ (*i*: ECL molar fraction, *j*: GVL molar fraction) of monomer mixtures in respect to obtained polymers for each polymer. GPC chromatograms of five monomers/polymer mixtures with known composition gave desired values (Fig C in S1 File). [*M*]_*eq*_ were calculated as [28]:

$${\left[M\right]}_{eq}={\left[M\right]}_{0}\frac{\beta_{i,j}A_{m}}{A_{p}+\beta_{i,j}A_{m}}.$$ (1)

In this equation, [*M*]_0_ is the starting overall concentration, while *A*_*m*_ and *A*_*p*_ are areas under peaks of UV traces, for monomer and polymer respectively (Fig D in S1 File).

[*M*]_*eq*_ were validated by comparing values of predicted and experimental *M*_*n*_ values. Predicted *M*_*n*_ values were calculated with the following equation:

$$M_{n}=\bar{M_{m}}\;\frac{{\left[M\right]}_{0}-{\left[M\right]}_{eq}}{{\left[I\right]}_{0}}+M_{I},$$ (2)

where $\bar{M_{m}}$ is the average molecular weight of monomers, [*I*]_0_ is the molar concentration of mPEG (0.1 mol L^−1^) and *M*_*I*_ is the molecular weight of mPEG (550 g mol^−1^).

Starting from the fundamental definition of the free Gibbs energy of polymerization Δ*G*_*p*_ = Δ*H*_*p*_ − *T*Δ*S*_*p*_, the Dainton-Ivin’s equation [29] assumes [*M*]_*eq*_ = exp[−Δ*G*/*RT*] [30] thus relating [*M*]_*eq*_ with temperature:

$$\text{ln}{\left[M\right]}_{eq}=\frac{\Delta H_{p}}{RT}-\frac{\Delta S_{p}}{R},$$ (3)

where Δ*H*_*p*_ is the enthalpy of polymerization, Δ*S*_*p*_ is the entropy of polymerization, *R* is the ideal gas constant (8.314 J mol^−1^ K^−1^). In our experiments, the degree of polymerization was comprised between 52 and 83, then Eq (3) did not need corrections. Ceiling temperatures *T*_*c*_ derived from Eq (3) assuming [*M*]_*eq*_ = [*M*]_0_. The linear fitting of ln[*M*]_*eq*_ *vs* 1/*T* gave the desired values of Δ*H*_*p*_ and Δ*S*_*p*_.

Macromolecular composition, calculated from ^1^H-NMR spectra, did not vary with reaction temperature, confirming that the thermodynamic equilibrium was reached after selected times.

[*ECL*]_*eq*_ and [*GVL*]_*eq*_ were estimated from experimental *M*_*n*_ and macromolecular composition, resulting:

$${\left[ECL\right]}_{eq}=\frac{N_{ECL,0}-N_{ECL,p}}{V_{tot}},$$ (4)

$${\left[GVL\right]}_{eq}=\frac{N_{GVL,0}-N_{GVL,p}}{V_{tot}},$$ (5)

where *N*_*ECL*,0_ and *N*_*GVL*,0_ are ECL and GVL moles in the starting reaction mixtures and *N*_*ECL*,*p*_ and *N*_*GVL*,*p*_ are ECL and GVL moles polymerized. Estimated values were validated by comparing predicted and experimental Δ*H*_*p*_ and Δ*S*_*p*_. Predicted thermodynamic parameters were calculated as:

$$\Delta H_{p}=\sum_{i}x_{i}\Delta H_{p,i},$$ (6)

$$\Delta S_{p}=\sum_{i}x_{i}\Delta S_{p,i}.$$ (7)

### ROP kinetics

Kinetic rates and monomer reactivity ratios were calculated from data obtained from low-conversion ROPs. Kinetic rates derived from fitting of experimental values of *M*_*n*_ *vs*. time. Reactivity ratios were calculated from the copolymerization kinetic equation (more details in SI):

$$\frac{d[ECL]}{d[GVL]}=(\frac{r_{1}\left[ECL\right]+\left[GVL\right]}{\left[ECL\right]+r_{2}\left[GVL\right]})\frac{\left[ECL\right]}{\left[GVL\right]}.$$ (8)

Solving Eq (8) we can calculate reactivity ratios and gather information about monomer distribution along the polymer chain.

### Non-isothermal ROP

DSC analysis provided information on kinetic rate parameters [31, 32]. We assumed the following reaction rate model:

$$\frac{d\alpha }{dt}=k\left(T\right)\cdot {\left(1-\alpha \right)}^{n}=A\text{exp}(-\frac{E_{a}}{RT})\cdot {\left(1-\alpha \right)}^{n}.$$ (9)

**Fig 2:**
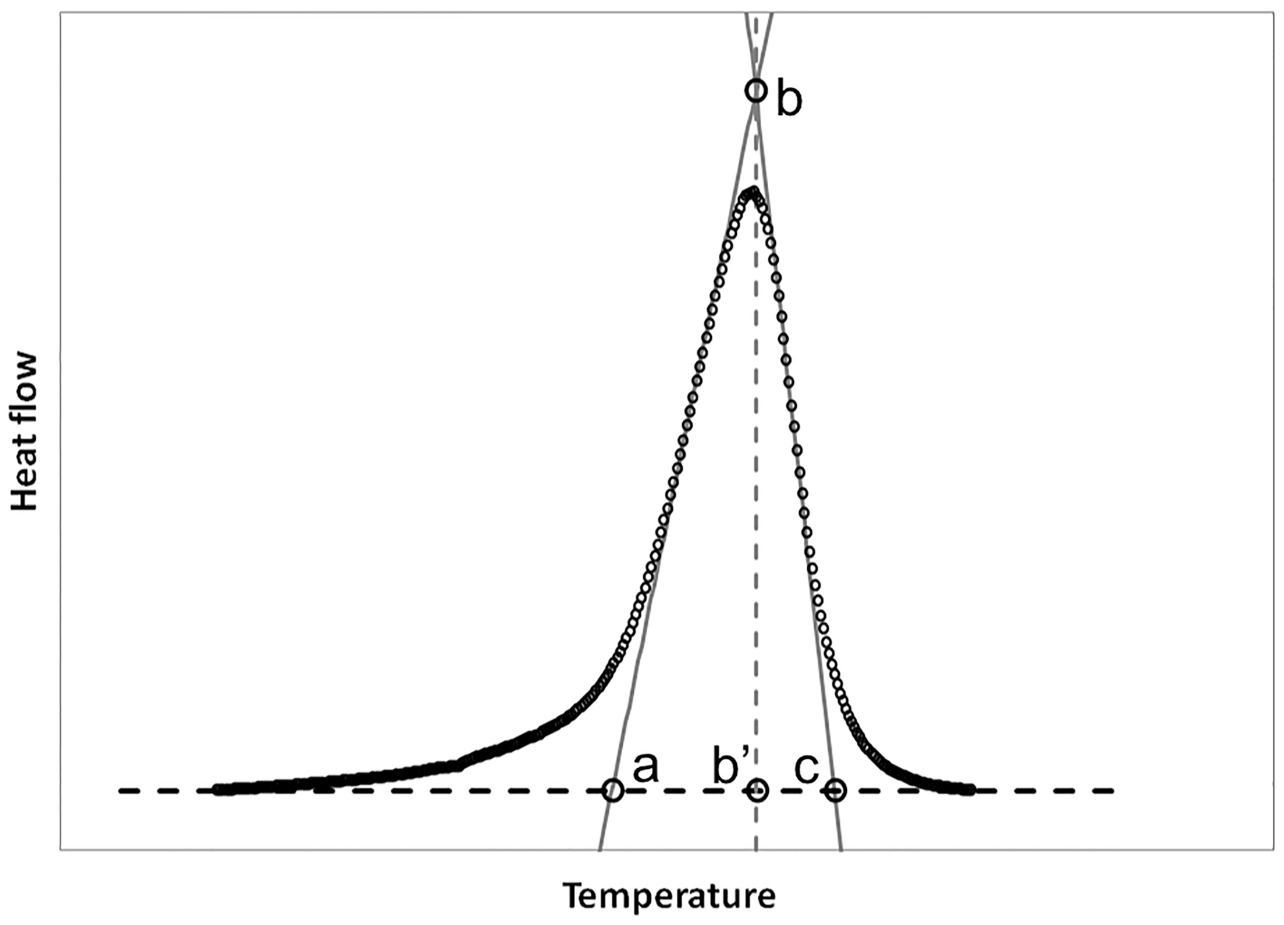
Characteristic temperatures: a onset temperature, b peak temperature, c offset temperature, and b’ projection of b; values were used to calculate *S*.

In this equation, *k*(*T*) is the temperature-dependent kinetic constant, *A* is the pre-exponential factor, *E*_*a*_ is the apparent activation energy (J mol^−1^), *T* is the absolute temperature (K), and *n* is the reaction order. The reaction order *n* is an estimation of reactant concentration effect on the reaction rate. Eq (9) is called *n*th order model and it is often used for the empirical description of heterogeneous processes affected by diffusion processes; it is derived from the Šesták–Berggren equation [33] *dα*/*dt* = *k*(*T*)*α*^*m*^(1 − *α*)^*n*^(−*ln*(1−*α*))^*p*^ setting *m* = *p* = 0. This generic model was selected to compute the unknown *n*th reaction order. Eq (9) contains three unknown parameters (*A*, *E*_*a*_ and *n*) while the triplet *T*, *α* and d*α*/d*t* was recorded by DSC. Unknown variables of Eq (9) were calculated with a multi-linear regression. Preliminary values of *n*, obtained from peak shapes with a geometric method, were used as first-attempt inputs. Assuming *n* constant over time, the shape index *S* was calculated from the degree of asymmetry of the peak as (Fig 2):

$$S=\frac{c-b^{\prime }}{b^{\prime }-a},$$ (10)

and *n* was then obtained as:

$$n=1.26\;\sqrt[{}]{S}$$ (11)

Values of *α* were normalized on the actual conversion calculated from GPC data. Tests were performed in triplicate.

## Results and discussion

### ROP thermodynamic

**Fig 3:**
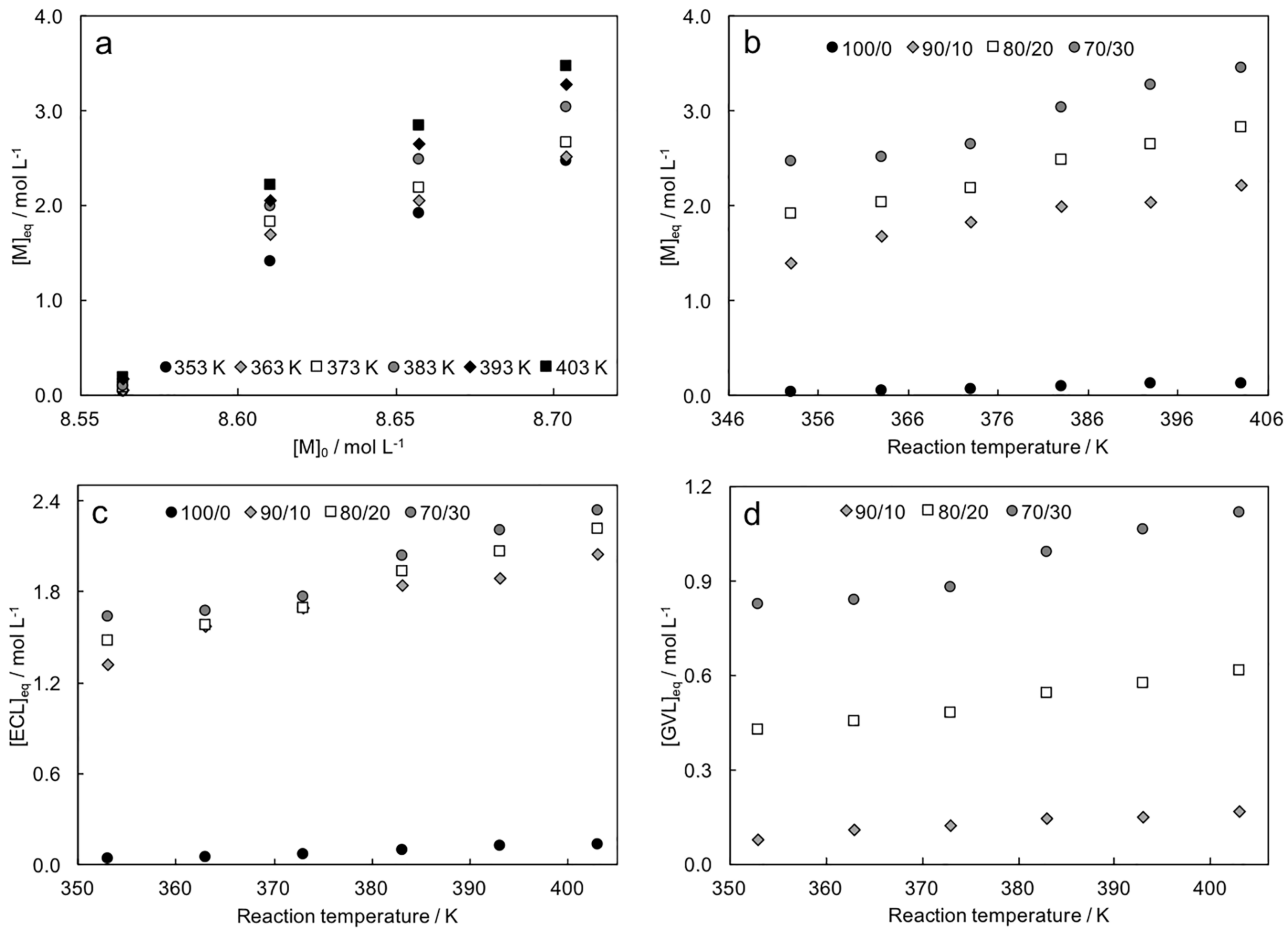
Thermodynamic equilibrium concentration [*M*]_*eq*_ *vs*. starting monomer concentration [*M*]_0_ at different temperatures (experimental data, from Eq 4); b) [*M*]_*eq*_ *vs*. reaction temperature for different ECL/GVL ratios in the starting monomer mixture; c) calculated [*ECL*]_*eq*_ *vs*. reaction temperature for different ECL/GVL ratios in the starting monomer mixture; d) calculated [*GVL*]_*eq*_ *vs*. reaction temperature for different ECL/GVL ratios in the starting monomer mixture; monomer ratios are indicated in figure legend.

Theoretical values of [*M*]_*eq*_ were considerably higher in copolymers than in material 100/0 (Table 2). This was due to the inhibition exerted by GVL monomer on the reaction. The value of [*M*]_*eq*_ for homopolymer 100/0 was closely in agreement with that reported in the literature for poly(*ϵ*-caprolactone) [5], while comparative data for copolymers were not available. [*M*]_*eq*_ increased with the increase of the starting GVL concentration in the monomer feed, indicating that ROP inhibition can be attributed to this monomer. The linear correlation between [*M*]_*eq*_ and [*M*]_0_ in copolymers (Fig 3a) confirmed that GVL affected the ROP mechanism. Reaction temperature influenced monomer equilibrium only in copolymer synthesis, while its effect was negligible in homopolymer reaction (Fig 3b). We combined GPC and ^1^H-NMR data to obtain a theoretical evaluation of single-monomer equilibrium concentration, [*ECL*]_*eq*_ and [*GVL*]_*eq*_ (Table 2). Single-monomer equilibrium concentration increased with decreasing ECL/GVL ratio. [*ECL*]_*eq*_ values in copolymerization were significantly higher than that calculated in material 100/0 (Fig 3c). This observation confirmed that GVL hindered the reaction, decreasing the ability of ECL to react via a ROP scheme. [*ECL*]_*eq*_ values were 25-30% the ECL concentration in reaction feeds ([*ECL*]_0_ = 7.7, 6.9 and 6.0 mol L^−1^ in copolymers 90/10, 80/20 and 70/30 respectively). Also [*GVL*]_*eq*_ depended on the starting molar composition of the reaction feed (Fig 3d). Its values linearly increased as the GVL concentration in reaction feeds increased. Equilibrium values were close to the starting concentrations (0.9, 1.8 and 2.7 mol L^−1^), indicating low reactivity of this monomer.

**Table 2:** [*M*]_*eq*_ (Eq (1)), calculated [*ECL*]_*eq*_ and [*GVL*]_*eq*_ (Eqs (4) and (5)), experimental (GPC) and calculated (Eq (2)) *M*_*n*_; values of *M*_*n*_ are referred to the polyester block, polydispersity index *PDI* calculated as the ratio *M*_*w*_/*M*_*n*_ from GPC data; reported GPC data are the mean of a triplicate synthesis.

Feed mixture (ECL/GVL)	[*M*]_*eq*_ (mol L ^−1^)	Calc. [*ECL*]_*eq*_ (mol L ^−1^)	Calc. [*GVL*]_*eq*_ (mol L ^−1^)	Ave. *M*_*n*_ (g mol ^−1^)	Calc. *M*_*n*_ (g mol ^−1^)	Calc. *PDI*
100/0	0.08	0.08	-	9900 ± 50	10200	1.25
90/10	1.85	1.67	0.19	7900 ± 450	8200	1.28
80/20	2.34	1.92	0.42	7300 ± 470	7700	1.26
70/30	2.90	2.11	0.78	6700 ± 510	7100	1.23

Calculated and experimental *M*_*n*_ values were in reasonable agreement (Table 2). While *M*_*n*_ value for material 100/0 was similar to the theoretical value (10 kDa) fixed in reaction setup, *M*_*n*_ values calculated for copolymers showed strong differences. This can be explained considering that GVL reactivity lowered the overall reaction yield while the theoretical starting value of the desired molecular weight was calculated hypothesizing the complete monomers conversion. The data can be used as reference to correct the reaction recipe if higher molecular weights are requested.

**Fig 4:**
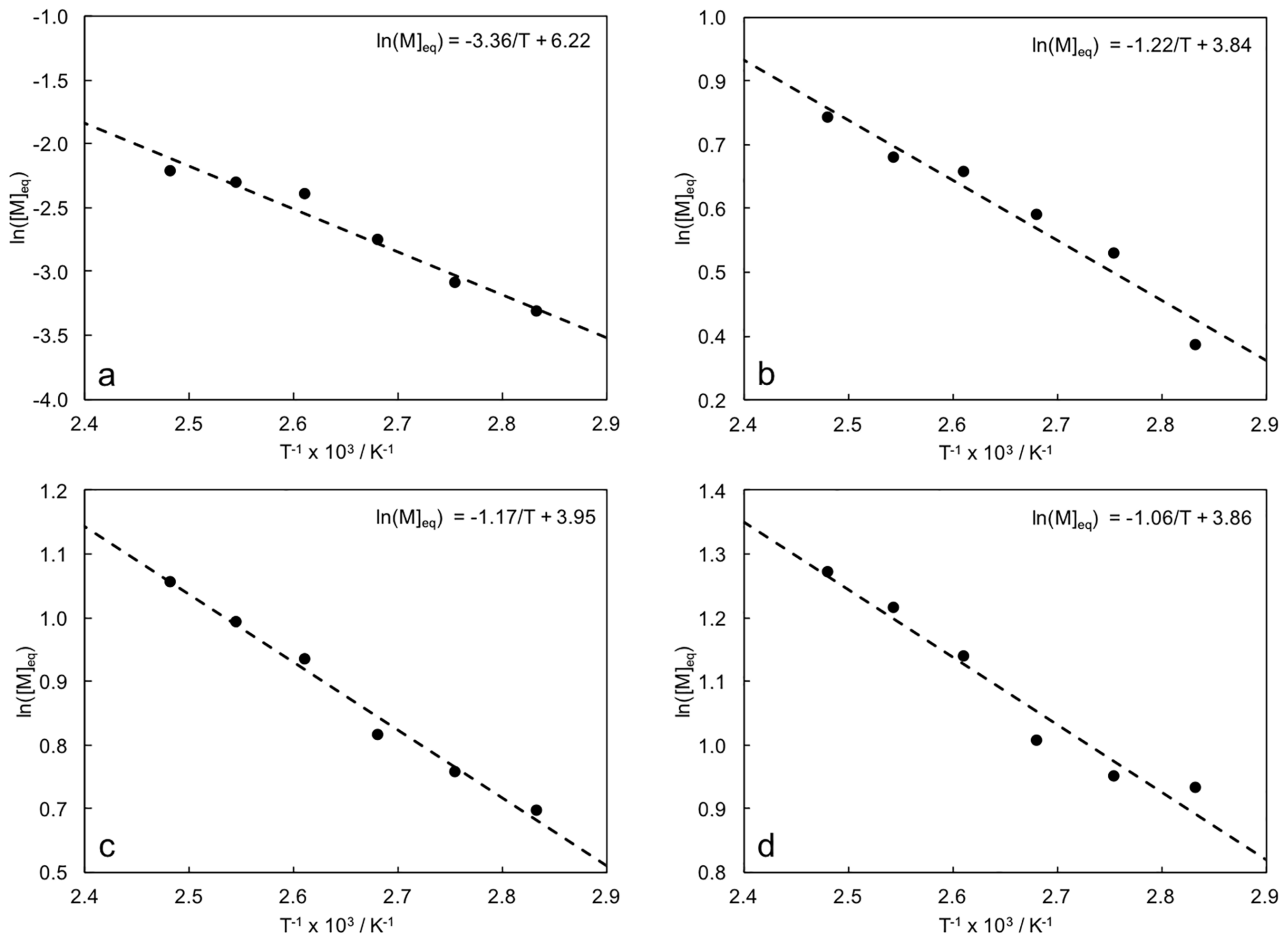
Linear fitting of ln([*M*]_*eq*_) *vs*. 1/*T*, material: a) 100/0, b) 90/10, c) 80/20 and d) 70/30; fitting equations are indicated for each material.

Trends of [*M*]_*eq*_ by varying *T* enabled the calculation of thermodynamic parameters (Fig 4 and Table 3), which were used to evaluate ceiling temperatures via the Dainton-Ivin equation (Eq (3)). While Δ*S*_*p*_ were not affected by the composition of the reaction feed, Δ*H*_*p*_ linearly increased with the GVL concentration. Calculated values indicated that major effects on ROP thermodynamics were given by the contribution of the reaction enthalpy. Higher Δ*H*_*p*_ values entailed higher Δ*G*_*p*_ and thus a lower aptitude to induce ROP. The results obtained for homopolymer 100/0 were close to those reported in the literature [34], while comparative data for copolymers were not available. Calculated values of *T*_*c*_ for the homopolymer was in agreement with the literature [35]. Values of *T*_*c*_ linearly decreased as the ECL/GVL ratio decreased, reducing the upper limit of the temperature range in which this ROP can occur.

**Table 3:** Molar enthalpy Δ*H*_*p*_ and entropy Δ*S*_*p*_ of polymerization, *R*^2^ of linear fitting of experimental data, *T*_*c*_ from Eq (3), calculated Δ*H*_*p*_ and Δ*S*_*p*_ from GPC and ^1^H-NMR data.

Feed mixture (ECL/GVL)	Δ*H*_*p*_ (kJ mol^−1^)	Δ*S*_*p*_ (J mol^−1^ K^−1^)	*R*^2^	*T*_*c*_ (K)	Calc. Δ*H*_*p*_ (kJ mol^−1^)	Calc. Δ*S*_*p*_ (J mol^−1^ K^−1^)
100/0	-27.9	-51.8	0.96	822	-27.9	-51.8
90/10	-10.2	-32.0	0.95	723	-10.4	-29.9
80/20	-9.7	-32.8	0.98	653	-9.8	-29.0
70/30	-8.8	-32.1	0.95	624	-8.9	-27.4

### ROP kinetics

Reaction rates indicate the speed at which monomers are consumed by the polymerization process. In addition to thermodynamic effects of the GVL monomer on this ROP, the kinetic analysis of copolymerization highlighted additional effects on ROP feasibility. Kinetic rates evaluated from residual monomer concentrations in low-conversion reactions (Table 4) confirmed that GVL affects ROP kinetics, slowing down the reaction. Calculated ECL consumption rate was significantly lower in copolymerization than that calculated in material 100/0. Decreasing trends for both ECL and GVL kinetic rates were registered as the ratio ECL/GVL decreased.

**Table 4:** Kinetic constant *k*, reaction order *n* and *R*^2^ from residual monomer analysis in low-conversion reactions.

Feed mixture (ECL/GVL)	Monomer	*k*(min^−1^)	*n*	*R*^2^
100/0		6.44⋅10^−3^	1.17	0.95
90/10	ECL	3.89⋅10^−3^	1.12	0.93
	GVL	4.65⋅10^−3^		0.94
80/20	ECL	3.05⋅10^−3^	1.14	0.96
	GVL	2.88⋅10^−3^		0.96
70/30	ECL	2.85⋅10^−3^	1.15	0.98
	GVL	2.53⋅10^−3^		0.98

The order of reaction *n* indicates how fast the concentration of reactants falls while the reaction proceeds. For this ROP, calculated values were close to unity in all cases and did not depend on monomer feed compositions. This indicates that reactions proceeded with a stepwise mechanism.

Reactivity ratios, calculated as reported in SI, were *r*_1_ = 0.65 ± 0.06 and *r*_2_ = 0.44 ± 0.02. These values indicated a slightly greater tendency of [−*ECL*^•^] to add the ECL monomer instead of the GVL, but similar reactivity ratios, both lower than 1, ensured the occurrence of the copolymerization, excluding a strong tendency of activated monomers to form homopolymers. Reactivity ratios product corroborate this conclusion. Their product was close to 0.3, indicating the aptitude of both monomers to form alternating or random copolymers [36]. However, lower amounts of GVL in the reaction feed did not allow formation of a perfectly alternating copolymer, as a random configuration is more probable under these conditions.

### Non-isothermal ROP

Non-isothermal DSC by varying heating rate provided data on kinetic rate parameters and defined lower values of temperature ranges to obtain significant polymerization rates. The unimodal distribution of DSC thermograms (Fig Ea in S1 File) revealed that the reaction kinetics was not affected by the molecular weight of growing polymer chains. Polymerization proceeded with equivalent chain-growth events, excluding the occurrence of side-reactions (e.g. trans-esterifications or inter- and intra- molecular reactions). The absence of significant side-reactions ensured that final products were linear macromolecules. The observation of equivalent chain-growth events confirmed our previous hypothesis, based on reactivity ratios calculations, which indicated a non-preferential tendency of activated monomers to add ECL or GVL. Monomer conversion *α* (Fig Eb in S1 File) and reaction rate d*α*/d*t* (Fig Ec in S1 File) traces shifted towards higher temperatures as the ECL/GVL ratio decreased. Final monomer conversion lowered in copolymers containing higher amounts of GVL. d*α*/d*t* curves were fitted, providing *E*_*a*_, *n* and *A*. Peak height increased as the heating rate increased, indicating faster polymerization rates.

**Fig 5:**
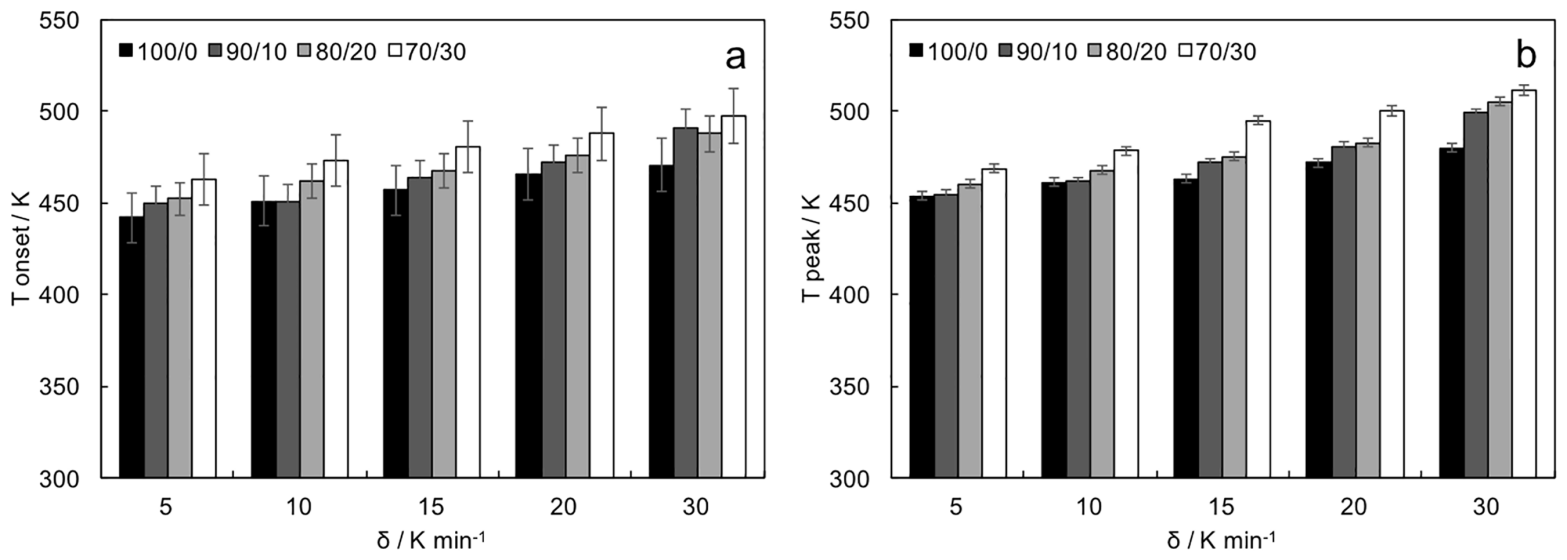
a) Onset and b) peak temperatures in non-isothermal polymerizations at different heating rates, as a function of the ECL/GVL ratio in the reaction feed.

Characteristic temperatures were calculated from DSC traces. Onset (Fig 5a) and peak (Fig 5b) temperatures were affected by heating rate and ECL/GVL ratio. Usually, onset temperatures should be not dependent on the heating rate. As for the ROP of ECL [37], we hypothesized that Sn(Oct)_2_ catalyst provided a coordination-insertion mechanism, which is governed by kinetics and thus by the temperature. Onset temperatures identified the lowest value to obtain significant reaction rates. At onset temperature, copolymerization was thermodynamically possible, but kinetics was too slow to be estimated. Peak temperatures identified the conditions to obtain the higher reaction rates. Values increased in copolymers with higher GVL amounts.

From non-isothermal ROP we obtained reaction orders and activation energies. Commonly, *E*_*a*_ is calculated with semi-empirical methods, based on maximum reaction rate or isoconversional maps. Although these methods have been used to study ROP of ECL [38–41], they present some restrictions that may limit their reliability. For the reported case, we performed a mathematical modeling of experimental data, avoiding approximations or model restrictions. Results of multi-linear regression are compared with those obtained with semi-empirical models in SI.

**Fig 6:**
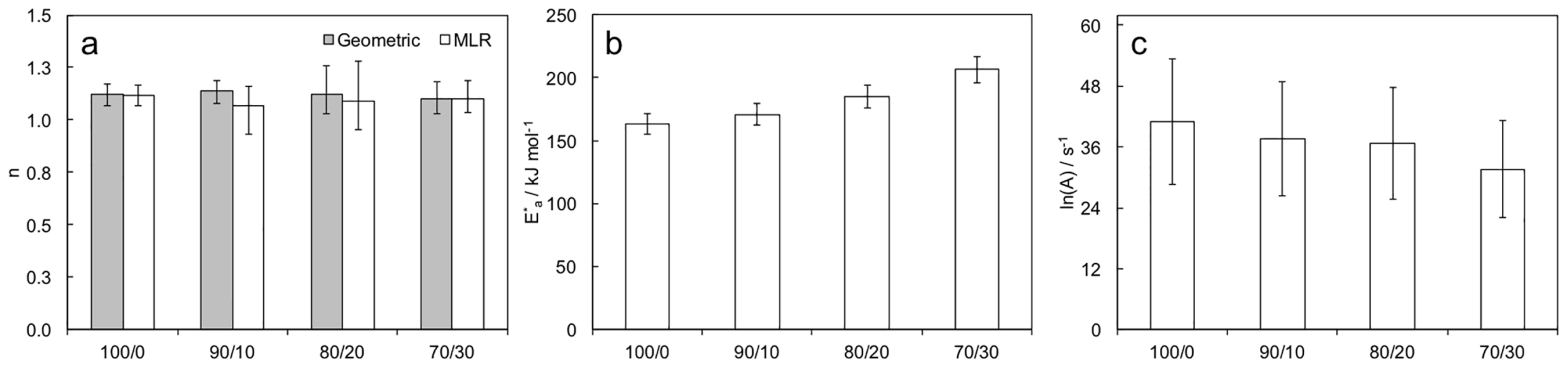
*n* from geometric method and multi-linear regression, *E*_*a*_ and ln(*A*) for analyzed ROPs.

Orders of reaction *n* were preliminary evaluated from curves d*α*/d*t* *vs* *T* with a geometric model. Values of *n* were close to 1.2 and not affected by feed mixture composition. Data obtained in non-isothermal analysis were in close agreement with those calculated in low-conversion reactions. Data were used as first tentative input of the multi-linear regression of d*α*/d*t* *vs* *T*. Multi-linear regression provided values of the triplet *E*_*a*_, *n* and ln(*A*). Values of *n* from multi-linear regression analysis confirmed those obtained with the geometric method (Fig 6a). *E*_*a*_ and ln(*A*) (Fig 6b and 6c) depended on the monomer feed composition, the first parameter increased while the second decreased as the ECL/GVL ratio decreased. Reported trends of *E*_*a*_ and ln(*A*) indicated that ROP kinetics was unfavorable in the presence of GVL.

## Conclusion

We demonstrated that ring-opening copolymerization of *γ*-valerolactone and *ϵ*-caprolactone is feasible under specific conditions of temperature and composition of the starting feed. By using different techniques, we identified limit conditions, in terms of temperature and monomer molar ratios, for such reaction. The combination of results from equilibrium, low-conversion and non-isothermal polymerizations enabled analyses of all major aspects related to kinetics and thermodynamics of this specific copolymerization process by varying the starting monomer feeds. We found that both kinetics and thermodynamics govern copolymerization and related parameters are affected by the amount of GVL used for the reaction. Equilibrium monomer concentrations and enthalpy of polymerization increased as the GVL concentration increased in the reaction feed. This indicates the existence of a thermodynamic upper limit in the concentration of this monomer. The upper monomer concentration limit explains why it was not possible to obtain homopolymers from GVL with high molecular weights with the proposed approach. The increase of reaction temperature caused the same effect on equilibrium monomer concentration, leading to a linear decrease of the ceiling temperature with the ECL/GVL ratio. Ceiling temperatures represent the upper temperature limit for this ROP, indicating the existence of a temperature range for copolymerization. Non-isothermal analysis identified the onset temperature providing significant copolymerization ratios. These results fix a lower temperature limit for the proposed ROP. Also, onset temperatures increased as the ECL/GVL ratio decreased, restricting the optimal range of temperatures for copolymerization. Overall monomer conversion was limited by the increasing GVL fraction, thus confirming that copolymerization was hampered in the presence of this monomer. While the reaction order *n* was not affected by monomer concentrations, trends of apparent activation energy and pre-exponential factor of kinetic constants confirmed the effect of GVL, which limited the reaction rate. Reactivity ratios were both smaller than 1 and their product suggested the formation of random copolymers.

Very few studies reported the synthesis of *γ*-lactone-based polyesters, probably due to the difficult ROP of five-membered rings, but their properties seem to be promising in several aspects, like degradation kinetics, mechanical properties and biocompatibility, even thought some preliminary studies on reaction conditions are needed to perform a controlled polymerization of *γ*-lactones. This paper clarifies the effects of reaction parameters on this specific copolymerization, and provides a guideline for the optimization of different ROPs in the presence of poorly reactive lactones.

## Supporting information

S1 File NMR analysis; calculation of [*M*]_*eq*_; calculation of reactivity ratios; non-isothermal DSC analysis. Fig A, ^1^H-NMR spectra of materials; 1: 100/0, 2: 90/10, 3: 80/20, 4: 70/30. Fig B, ^13^C-NMR spectra of materials; 1: 100/0, 2: 90/10, 3: 80/20, 4: 70/30. Fig C, Calibration curves used for the calculation of *β*_*i*,*j*_ coefficients; a) 100/0, b) 90/10, c) 80/20 and d) 70/30. Fig D, GPC traces of crude samples withdrawn at the end of equilibrium reactions performed at different temperatures; a) 100/0, b) 90/10, c) 80/20 and d) 70/30. Fig E, DSC analysis: a) thermograms of normalized heat flow (W g^-1^), b) *α* and c) d*α*/d*t* against T at different heating rates. Fig F, Friedman plots (monomer conversion range: 0.2–0.9); a) 100/0, b) 90/10, c) 80/20 and d) 70/30. Fig G, KAS plots (monomer conversion range: 0.2–0.9); a) 100/0, b) 90/10, c) 80/20 and d) 70/30. Fig H, OFW plots (monomer conversion range: 0.2–0.9); a) 100/0, b) 90/10, c) 80/20 and d) 70/30. Fig I, *E*_*a*_ *vs* *α* from Friedman, KAS and OFW methods; a) 100/0, b) 90/10, c) 80/20 and d) 70/30. Fig J, *E*_*a*_ evaluated by a) maximum reaction rate temperatures, b) isoconversional methods and c) numerical methods. Table A, *M*_*n*_ (Da) and *α* obtained from equilibrium polymerizations at different temperatures; values of *M*_*n*_ are referred to the polyester block. Table B, Calculated thermodynamic parameters for each monomer. Table C, *M*_*n*_ (Da) and [*M*]_*res*_ (mol L^−1^) from low-conversion polymerization at different times; values of *M*_*n*_ are referred to the polyester block. Table D, *M*_*n*_ (Da) and *α* from non-isothermal ROP at different heating rates; values of *M*_*n*_ are referred to the polyester block.
(PDF)
