# Supplementary material for: Ring-opening copolymerization thermodynamics and kinetics of γ-valerolactone/ϵ-caprolactone
Source: PLoS One. 2018 Jun 21;13(6):e0199231. doi: 10.1371/journal.pone.0199231 (PMC6013151; doi:10.1371/journal.pone.0199231)
Supplement: S1 File — Fig A, 1H-NMR spectra of materials; 1: 100/0, 2: 90/10, 3: 80/20, 4: 70/30. Fig B, 13C-NMR spectra of materials; 1: 100/0, 2: 90/10, 3: 80/20, 4: 70/30. Fig C, Calibration curves used for the calculation of βi,j coefficients; a) 100/0, b) 90/10, c) 80/20 and d) 70/30. Fig D, GPC traces of crude samples withdrawn at the end of equilibrium reactions performed at different temperatures; a) 100/0, b) 90/10, c) 80/20 and d) 70/30. Fig E, DSC analysis: a) thermograms of normalized heat flow (W g-1), b) α and c) dα/dt against T at different heating rates. Fig F, Friedman plots (monomer conversion range: 0.2–0.9); a) 100/0, b) 90/10, c) 80/20 and d) 70/30. Fig G, KAS plots (monomer conversion range: 0.2–0.9); a) 100/0, b) 90/10, c) 80/20 and d) 70/30. Fig H, OFW plots (monomer conversion range: 0.2–0.9); a) 100/0, b) 90/10, c) 80/20 and d) 70/30. Fig I, Ea vs α from Friedman, KAS and OFW methods; a) 100/0, b) 90/10, c) 80/20 and d) 70/30. Fig J, Ea evaluated by a) maximum reaction rate temperatures, b) isoconversional methods and c) numerical methods. Table A, Mn (Da) and α obtained from equilibrium polymerizations at different temperatures; values of Mn are referred to the polyester block. Table B, Calculated thermodynamic parameters for each monomer. Table C, Mn (Da) and [M]res (mol L−1) from low-conversion polymerization at different times; values of Mn are referred to the polyester block. Table D, Mn (Da) and α from non-isothermal ROP at different heating rates; values of Mn are referred to the polyester block. (PDF) [file pone.0199231.s001.pdf]

Ring-opening copolymerization thermodynamics and kinetics of γ-valerolactone/ϵ-caprolactone

Mariacristina Gagliardi[1] and Angelo Bifone[2]

[1]Center for Micro Bio-Robotics @SSSA, Istituto Italiano di Tecnologia, viale Rinaldo Piaggio, 34, 56025, Pontedera, Italy
[2]Center for Neuroscience and Cognitive Systems @UNITN, Istituto Italiano di Tecnologia, Corso Bettini 31, 38068 Rovereto, Italy

Supporting Information

Contents

1 NMR analysis **3**

2 Calculation of $[M]_{eq}$ **4**
- 2.1 Calibration curves used for the calculation of $\beta_{i,j}$ coefficients . . 4
- 2.2 GPC traces used for the calculation of $[M]_{eq}$ 5

3 Calculation of reactivity ratios **6**

4 Non-isothermal DSC analysis **6**
- 4.1 Maximum reaction rate at different heating rates (Kissinger, Ozawa) 7
- 4.2 Isoconversional maps (Friedman, KAS and OFW) 7
- 4.3 Coats-Redfern integral method (CR) 8
- 4.4 Comparison of results 8

List of Figures

Figure A: ^{1}H-NMR spectra of materials; 1: 100/0, 2: 90/10, 3: 80/20, 4: 70/30. 3

Figure B: ^{13}C-NMR spectra of materials; 1: 100/0, 2: 90/10, 3: 80/20, 4: 70/30. 3

Figure C: Calibration curves used for the calculation of $\beta_{i,j}$ coefficients; a) 100/0, b) 90/10, c) 80/20 and d) 70/30. 4

Figure D: GPC traces of crude samples withdrawn at the end of equilibrium polymerizations performed at different temperatures; a) 100/0, b) 90/10, c) 80/20 and d) 70/30. . . 5
Figure E: DSC analysis: a) thermograms of normalized heat flow (W g^{-1}), b) α and c) dα/dt against T at different heating rates. 9
Figure F: Friedman plots (monomer conversion range: 0.2 - 0.9); a) 100/0, b) 90/10, c) 80/20 and d) 70/30. 10
Figure G: KAS plots (monomer conversion range: 0.2 - 0.9); a) 100/0, b) 90/10, c) 80/20 and d) 70/30. 10
Figure H: OFW plots (monomer conversion range: 0.2 - 0.9); a) 100/0, b) 90/10, c) 80/20 and d) 70/30. 11
Figure I: E_a *vs* α from Friedman, KAS and OFW methods; a) 100/0, b) 90/10, c) 80/20 and d) 70/30. 11
Figure J: E_a evaluated by: a) maximum reaction rate temperatures, b) isoconversional methods and c) numerical methods. 12

List of Tables

Table A: M_n (Da) and α obtained from equilibrium polymerizations at different temperatures; values of M_n are referred to the polyester block. 13
Table B: Calculated thermodynamic parameters for each monomer. 13
Table C: M_n (Da) and residual molar concentration $[M]_{res}$ (mol L^{-1}) from low-conversion polymerization at different times; values of M_n are referred to the polyester block. 13
Table D: M_n (Da) and α from non-isothermal ROP at different heating rates; values of M_n are referred to the polyester block. 14

1 NMR analysis

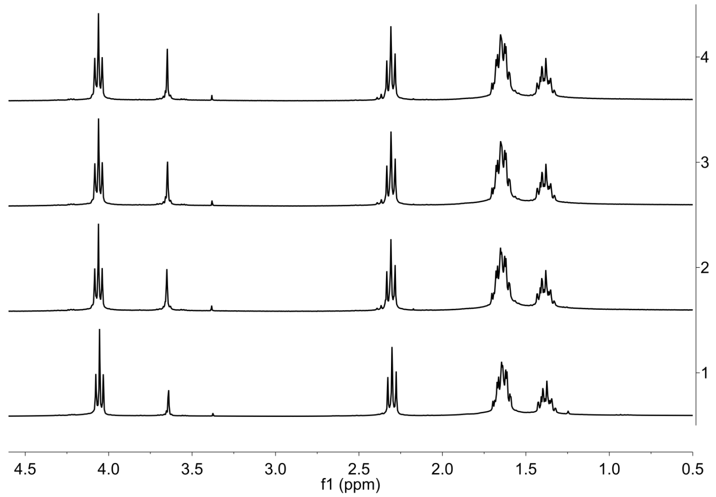

Figure A: ^{1}H-NMR spectra of materials; 1: 100/0, 2: 90/10, 3: 80/20, 4: 70/30.

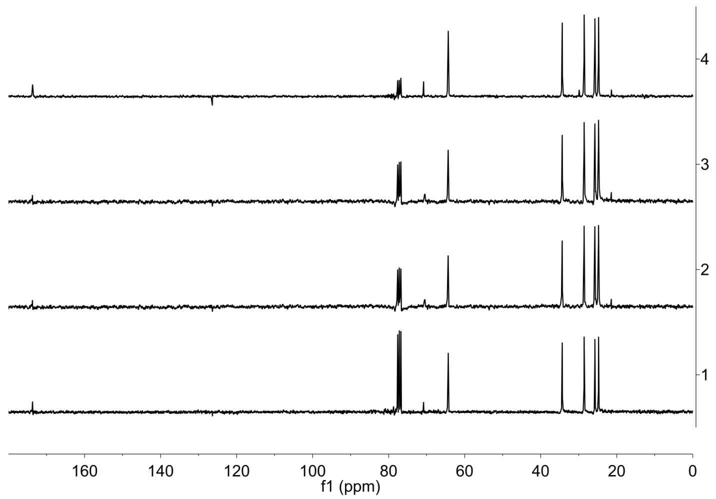

Figure B: ^{13}C-NMR spectra of materials; 1: 100/0, 2: 90/10, 3: 80/20, 4: 70/30.

2 Calculation of $[M]_{eq}$

2.1 Calibration curves used for the calculation of $\beta_{i,j}$ coefficients

Calibration curves were obtained from the analysis of five polymer/monomer mixtures with known concentration.
In C Fig, RI_M and RI_P are peak values of the RI signal attributed to monomer and polymer peaks respectively, n_M and n_P are the number of moles introduced in reference mixtures of monomers and polymers respectively. Values of $\beta_{i,j}$ coefficients are given by the slope of the linear fitting.

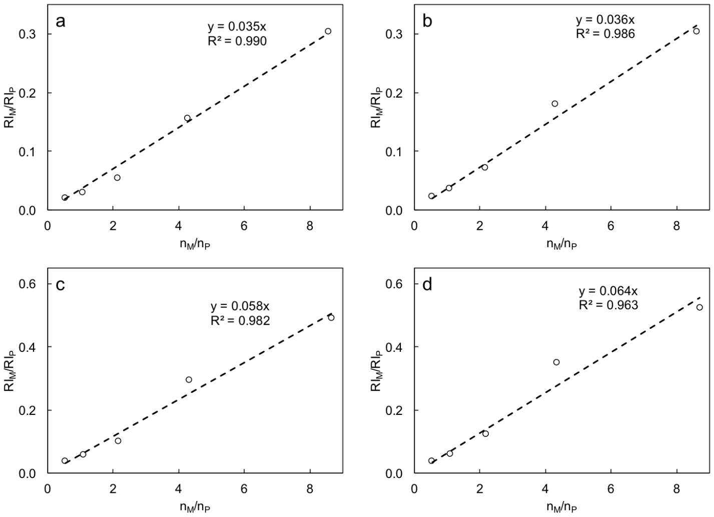

Figure C: Calibration curves used for the calculation of $\beta_{i,j}$ coefficients; a) 100/0, b) 90/10, c) 80/20 and d) 70/30.

2.2 GPC traces used for the calculation of $[M]_{eq}$

Acquired chromatograms on crude samples withdrawn at the end of equilibrium polymerization, performed at different temperatures in the range from 80 °C to 130 °C. Reaction times depended on the imposed temperature, as specified in the text.

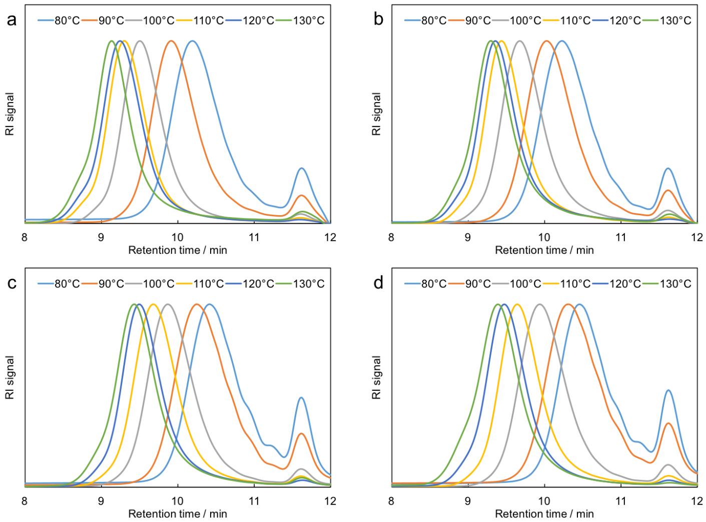

Figure D: GPC traces of crude samples withdrawn at the end of equilibrium polymerizations performed at different temperatures; a) 100/0, b) 90/10, c) 80/20 and d) 70/30.

3 Calculation of reactivity ratios

Assuming that kinetic rates do not vary with the macromolecular chain length, consumption rates of ECL and GVL at a generic propagation step, expressed as molar concentrations, are described as:

$$-\frac{\mathrm{d}[ECL]}{\mathrm{d}t} = \left(k_{11}[-ECL^\bullet] + k_{21}[-GVL^\bullet]\right)[ECL], \tag{1}$$

$$-\frac{\mathrm{d}[GVL]}{\mathrm{d}t} = \left(k_{12}[-ECL^\bullet] + k_{22}[-GVL^\bullet]\right)[GVL], \tag{2}$$

where $[-ECL^\bullet]$ and $[-GVL^\bullet]$ are molar concentrations of active species. Taking the ratio:

$$\frac{\mathrm{d}[ECL]}{\mathrm{d}[GVL]} = \frac{k_{11}[-ECL^\bullet] + k_{21}[-GVL^\bullet]}{k_{12}[-ECL^\bullet] + k_{22}[-GVL^\bullet]} \cdot \frac{[ECL]}{[GVL]}, \tag{3}$$

and assuming the steady-state:

$$k_{12}[-ECL^\bullet][GVL] = k_{21}[-GVL^\bullet][ECL], \tag{4}$$

we obtain:

$$\frac{\mathrm{d}[ECL]}{\mathrm{d}[GVL]} = \left(\frac{\frac{k_{11}}{k_{12}}[ECL] + [GVL]}{[ECL] + \frac{k_{22}}{k_{21}}[GVL]}\right)\frac{[ECL]}{[GVL]}. \tag{5}$$

Finally, introducing reactivity ratios $r_1 = \frac{k_{11}}{k_{12}}$ and $r_2 = \frac{k_{22}}{k_{21}}$ in Eq. (3) we obtain:

$$\frac{\mathrm{d}[ECL]}{\mathrm{d}[GVL]} = \left(\frac{r_1[ECL] + [GVL]}{[ECL] + r_2[GVL]}\right)\frac{[ECL]}{[GVL]}. \tag{6}$$

Eq. (6) allows calculating reactivity ratios.

4 Non-isothermal DSC analysis

DSC analysis estimates the instantaneous polymerization enthalpy ΔH_p, which is related to the instantaneous monomer conversion α as:

$$\alpha = \frac{\Delta H_p}{\Delta H_{p,tot}}, \tag{7}$$

where $\Delta H_{p,tot}$ is the overall enthalpy of polymerization. A generic expression for the reaction rate $\frac{\mathrm{d}\alpha}{\mathrm{d}t}$ is:

$$\frac{\mathrm{d}\alpha}{\mathrm{d}t} = kf(\alpha), \tag{8}$$

where $f(\alpha)$ depends on the reaction model and k is the Arrhenius kinetic constant:

$$k = A\exp\left(-\frac{E_a}{RT}\right). \tag{9}$$

A is the Arrhenius pre-exponential factor, E_a the activation energy, R the ideal gas constant, T the absolute temperature. When indirect analytical methods are used, like DSC, E_a is the apparent activation energy. To identify reaction parameters, a valid model for $f(\alpha)$ is $f(\alpha) = (1-\alpha)^n$. Introducing this function in Eq. (8) we obtain:

$$\frac{\mathrm{d}\alpha}{\mathrm{d}t} = A \exp\left(-\frac{E_a}{RT}\right) \cdot (1-\alpha)^n, \tag{10}$$

where n the reaction order. DSC thermograms, α and $\mathrm{d}\alpha/\mathrm{d}t$ are reported in E Fig.

Unknown parameters in Eq. (10) (A, E_a and n) are evaluated via semi-empirical methods based on: maximum reaction rate (Kissinger and Ozawa models), isoconversional maps (Friedman, Kissinger-Akahira-Sunose or KAS and Ozawa-Flynn-Wall or OFW models), integral methods (Coats-Redfern or CR model). Semi-empirical methods are simple and fast to apply but are based on assumptions that limit their reliability.

4.1 Maximum reaction rate at different heating rates (Kissinger, Ozawa)

Kissinger and Ozawa models are based on two assumptions: (1) α at the maximum reaction rate does not depend on heating rate, and (2) n is assumed equal to one. E_a derives from Kissinger and Ozawa plots.
Kissinger equation [1] is:

$$\frac{\delta}{T_p^2} = \ln\left(\frac{AR}{E_a}\right) - \frac{E_a}{RT_p}. \tag{11}$$

δ is the heating rate (K s^{-1}), T_p is the maximum reaction rate temperature. E_a derives from the slope of $\ln(\delta/T_p^2)$ *vs* $1/T_p$.
Ozawa equation [2] combined with the Doyle approximation [3] is:

$$\ln(\delta) = c - 1.052\frac{E_a}{RT_p}. \tag{12}$$

c is an empirical constant, the slope of $\ln(\delta)$ *vs* $1/T_p$ gives E_a.

4.2 Isoconversional maps (Friedman, KAS and OFW)

Isoconversional methods are based on the assumption that E_a is constant over the whole monomer conversion range.
Friedman equation derives from the differential isoconversional analysis [4]. Taking the logarithmic form of Eq. (10), we obtain:

$$\frac{\mathrm{d}\alpha}{\mathrm{d}t} = \ln(A) - \frac{E_a}{RT} + n\ \ln(1-\alpha). \tag{13}$$

The slope of $\ln(\mathrm{d}\alpha/\mathrm{d}t)$ *vs* $1/T$ gives E_a.
Isoconversional maps for KAS and OFW methods are obtained from Eq. (11) and Eq. (12) respectively, introducing values of T at fixed α.

4.3 Coats-Redfern integral method (CR)

Coats-Redfern integral method was developed in a generic form for n-order reaction kinetics [5]. Introducing $\delta = \mathrm{d}T/\mathrm{d}t$ and rearranging Eq. (10) we obtain:

$$\int_0^\alpha \frac{\mathrm{d}\alpha}{(1-\alpha)^n} = \frac{A}{\delta}\int_0^T \exp\left(-\frac{E_a}{RT}\right)\mathrm{d}T \tag{14}$$

The right-hand integral cannot be solved exactly but can be approximated obtaining:

$$\frac{1-(1-\alpha)^{1-n}}{1-n} = \frac{ART^2}{\delta E_a}\left(1-\frac{2RT}{E_a^*}\right)\exp\left(-\frac{E_a}{RT}\right) \tag{15}$$

From the multi-linear regression of $\ln \frac{1-(1-\alpha)^{1-n}}{T^2\ (1-n)}$ *vs* $1/T$ derive E_a and n.

4.4 Comparison of results

The analysis of reaction kinetics highlighted that (i) reaction rate was dependent on the heating rate and (ii) activation energy significantly vary with monomer conversion. Both phenomena indicated that semi-empirical methods are not valid for the analyzed ROP. Thus, generic models, which do not imply restrictions for the analysis, are more reliable.
Semi-empirical models gave similar values of E_a (Ja-b Figs). On the contrary, data calculated with multi-linear regression and integral methods were significantly different. On the basis of reported results, we conclude that for this specific ROP we cannot assume any simplification and only an accurate numerical analysis gives significant results.

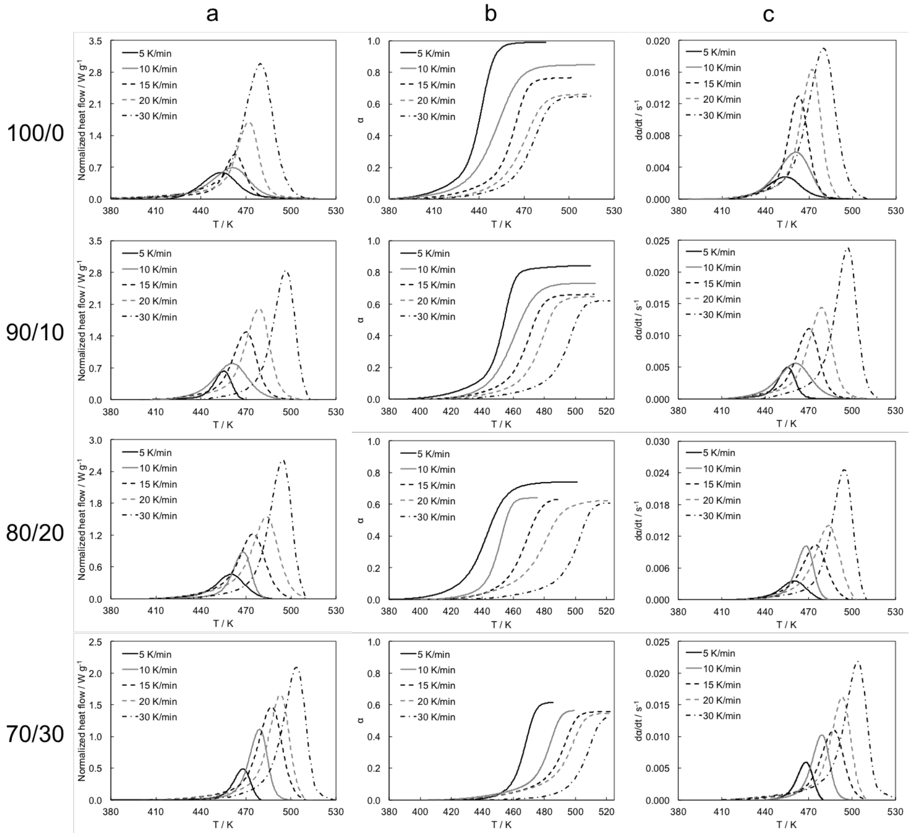

Figure E: DSC analysis: a) thermograms of normalized heat flow (W g^{-1}), b) α and c) dα/dt against T at different heating rates.

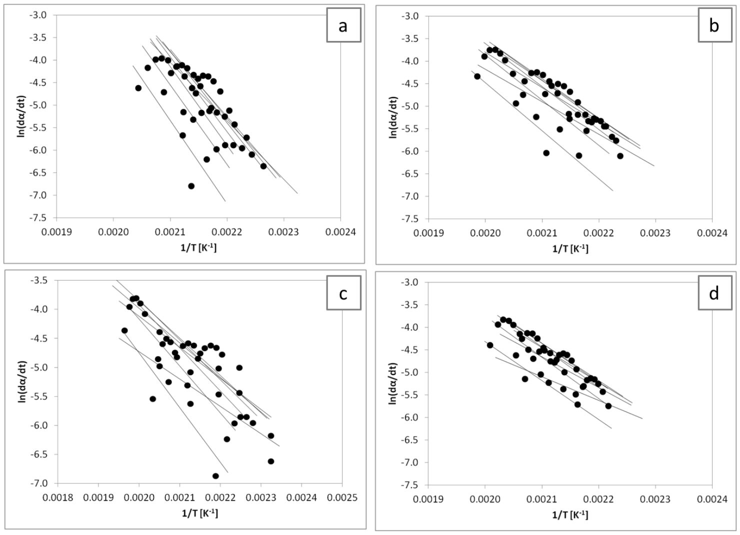

Figure F: Friedman plots (monomer conversion range: 0.2 - 0.9); a) 100/0, b) 90/10, c) 80/20 and d) 70/30.

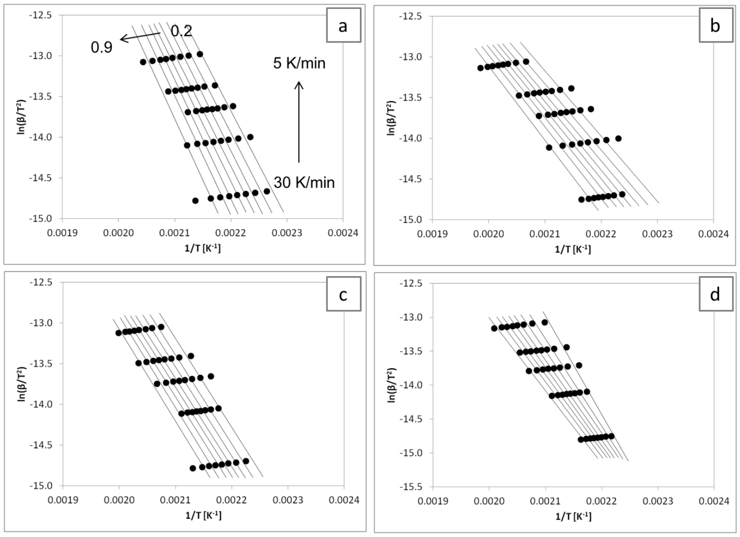

Figure G: KAS plots (monomer conversion range: 0.2 - 0.9); a) 100/0, b) 90/10, c) 80/20 and d) 70/30.

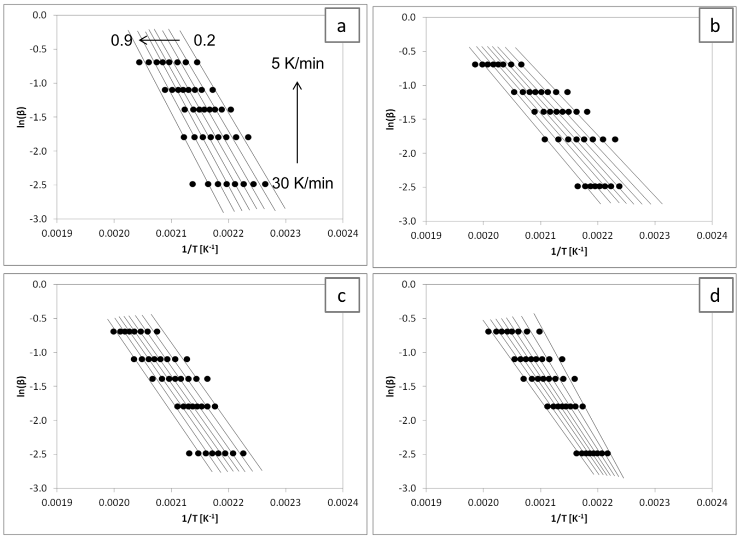

Figure H: OFW plots (monomer conversion range: 0.2 - 0.9); a) 100/0, b) 90/10, c) 80/20 and d) 70/30.

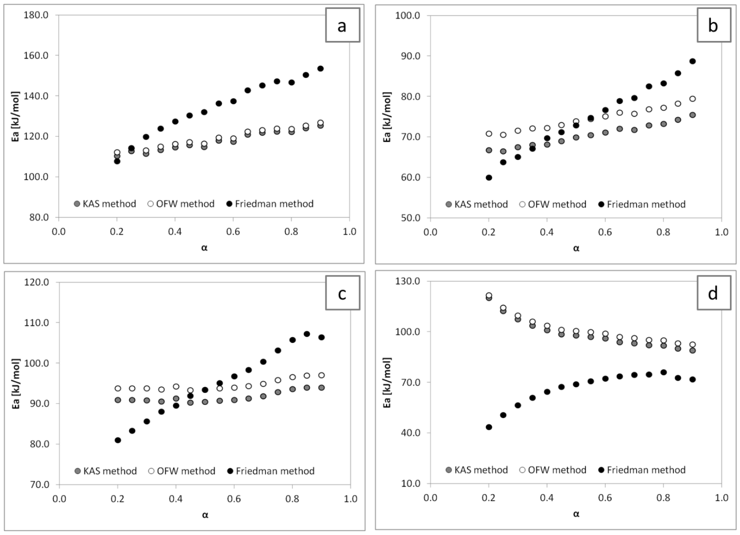

Figure I: E_a *vs* α from Friedman, KAS and OFW methods; a) 100/0, b) 90/10, c) 80/20 and d) 70/30.

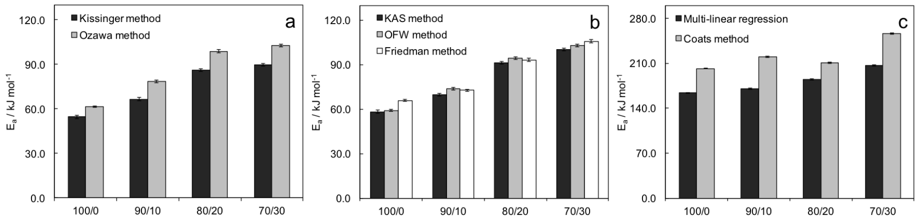

Figure J: E_a evaluated by: a) maximum reaction rate temperatures, b) isoconversional methods and c) numerical methods.

Table A: M_n (Da) and α obtained from equilibrium polymerizations at different temperatures; values of M_n are referred to the polyester block.

**T (K)**	**100/0**		**90/10**		**80/20**		**70/30**	
	M_n	α	M_n	α	M_n	α	M_n	α
353	9960	1.00	8390	0.84	7790	0.78	7150	0.72
363	9950	1.00	8080	0.81	7650	0.77	7100	0.71
373	9930	0.99	7930	0.79	7500	0.75	6950	0.70
383	9900	0.99	7750	0.78	7180	0.72	6550	0.66
393	9890	0.99	7690	0.77	7000	0.70	6300	0.63
403	9880	0.99	7500	0.75	6800	0.68	6100	0.61

Table B: Calculated thermodynamic parameters for each monomer.

**Feed composition (ECL/GVL)**	$\beta_{i,j}$	**ECL** ΔH_p	**ECL** ΔS_p	**GVL** ΔH_p	**GVL** ΔS_p
100/0	0.510	-29.7	-51.8	-	-
90/10	0.461	-9.7	-30.1	-17.0	-27.9
80/20	0.450	-10.0	-31.4	-8.8	-17.8
70/30	0.450	-9.2	-29.9	-7.9	-20.5

Table C: M_n (Da) and residual molar concentration $[M]_{res}$ (mol L^{-1}) from low-conversion polymerization at different times; values of M_n are referred to the polyester block.

**Time (min)**	**100/0**		**90/10**		**80/20**		**70/30**	
	M_n	$[M]_{res}$	M_n	$[M]_{res}$	M_n	$[M]_{res}$	M_n	$[M]_{res}$
15	785	7.85	734	7.93	494	8.20	451	8.28
30	1317	7.37	1093	7.60	708	8.00	633	8.11
45	2726	6.09	2041	6.73	1490	7.27	1343	7.43
75	3836	5.09	3007	5.84	2105	6.69	2209	6.61
120	4259	4.70	3445	5.44	2805	6.04	2994	5.87
180	5079	3.96	3937	4.99	3235	5.64	3599	5.30
240	8005	1.31	5163	3.86	5368	3.65	4519	4.43
300	9408	0.04	6731	2.41	5551	3.48	5602	3.41

Table D: M_n (Da) and α from non-isothermal ROP at different heating rates; values of M_n are referred to the polyester block.

**Heating rate (K min^{-1})**	**100/0**		**90/10**		**80/20**		**70/30**	
	M_n	α	M_n	α	M_n	α	M_n	α
5	9890	0.99	8430	0.83	7410	0.73	6140	0.59
10	8740	0.87	7310	0.72	6410	0.62	5630	0.54
15	7660	0.75	6610	0.64	6330	0.61	5580	0.53
20	6600	0.64	6470	0.63	6240	0.60	5470	0.52
30	6480	0.63	6820	0.66	6080	0.59	5210	0.49

References

[1] Kissinger HE. Variation of peak temperature with heating rate in differential thermal analysis. J Res Natl Bur Stand. 1956;57(4):217.

[2] Ozawa T. A New Method of Analyzing Thermogravimetric Data. Bull Chem Soc Jpn. 1965;38(11):1881–1886.

[3] Doyle CD. Kinetic analysis of thermogravimetric data. J Appl Polym Sci. 1962;6(19):120–120.

[4] Friedman HL. Kinetics of thermal degradation of char-forming plastics from thermogravimetry. Application to a phenolic plastic. J Polym Sci C. 2007;6(1):183–195.

[5] Coats AW, Redfern JP. Kinetic Parameters from Thermogravimetric Data. Nature. 1964;201(4914):68–69.
